# Supplementary material for: Gluten-Free Bread Enriched with Artichoke Leaf Extract In Vitro Exerted Antioxidant and Anti-Inflammatory Properties
Source: Antioxidants (Basel). 2023 Apr 1;12(4):845. doi: 10.3390/antiox12040845 (PMC10135093; doi:10.3390/antiox12040845)
Supplement: Supplementary file 1 [file antioxidants-12-00845-s001.zip › Supplementary_Tables_S1_S2.pdf]

Supplementary Table S1. Significantly different compounds (p-value <0.05; one way ANOVA test corrected by Tukey's procedure) found between gluten free bread fermented fecal batches after 20h (T20) of fermentation. Breads were differently labelled based on the use of sourdough (SB) or not (YB) and based on the artichoke extract (AE) addition.

Compound	f-value	p-value	FDR	Tukey's HSD
**Hexanal**	21.725	0.00033581	0.01151	SBAE-T20 vs SB-T20; YB-T20 vs SB-T20; YBAE-T20 vs SB-T20; YBAE-T20 vs SBAE-T20; YBAE-T20 vs YB-T20
**Oxirane, tetradecyl-**	20.817	0.00039016	0.01151	SBAE-T20 vs SB-T20; YB-T20 vs SBAE-T20; YBAE-T20 vs SBAE-T20
**1-Propanol**	14.274	0.0014125	0.02778	YB-T20 vs SB-T20; YBAE-T20 vs SB-T20
**Pentadecanal-**	10.766	0.0035052	0.039647	SBAE-T20 vs SB-T20; YB-T20 vs SBAE-T20; YBAE-T20 vs SBAE-T20
**Undecane**	10.392	0.0039133	0.039647	YB-T20 vs SB-T20; YBAE-T20 vs SB-T20; YB-T20 vs SBAE-T20; YBAE-T20 vs SBAE-T20
**Decane**	10.188	0.0041613	0.039647	YB-T20 vs SB-T20; YB-T20 vs SBAE-T20; YBAE-T20 vs YB-T20
**Hydrocinnamic acid**	9.544	0.0050841	0.039647	SBAE-T20 vs SB-T20; YB-T20 vs SBAE-T20; YBAE-T20 vs SBAE-T20
**3-Ethyl-3-methylheptane**	9.3703	0.0053759	0.039647	YB-T20 vs SB-T20; YB-T20 vs SBAE-T20; YBAE-T20 vs YB-T20
**Butanoic acid, ethyl ester**	8.3872	0.0074857	0.048452	SBAE-T20 vs SB-T20; YBAE-T20 vs SBAE-T20
**Benzaldehyde, 2,5-dimethyl-**	8.0225	0.0085254	0.048452	YB-T20 vs SBAE-T20; YBAE-T20 vs SBAE-T20
**Dodecane**	7.864	0.0090334	0.048452	YB-T20 vs SB-T20; YB-T20 vs SBAE-T20

Supplementary Table S2. Significantly different compounds (p-value <0.05; one way ANOVA test corrected by Tukey's procedure) found between gluten free bread fermented fecal batches after 42h (T42) of fermentation. Breads were differently labelled based on the use of sourdough (SB) or not (YB) and based on the artichoke extract (AE) addition.

Compound	f.value	p.value	FDR	Tukey's HSD
**Ethanol**	80.894	2.5197e-06	0.00013859	SBAE-T42 vs SB-T42; YB-T42 vs SB-T42; YBAE-T42 vs SB-T42; YBAE-T42 vs YB-T42
**1-Butanol**	61.47	7.2312e-06	0.00019886	SBAE-T42SB-T42; YB-T42SB-T42; YBAE-T42 vs SB-T42
**Hexanoic acid**	48.148	1.8269e-05	0.00032756	YB-T42 vs SB-T42; YBAE-T42 vs SB-T42; YB-T42 vs SBAE-T42; YBAE-T42 vs SBAE-T42; YBAE-T42 vs YB-T42
**Hydrocinnamic acid**	44.867	2.3823e-05	0.00032756	SBAE-T42 vs SB-T42; YBAE-T42 vs SB-T42; YB-T42 vs SBAE-T42; YBAE-T42 vs SBAE-T42
**Heptanoic acid**	38.077	4.3958e-05	0.00048354	SBAE-T42 vs SB-T42; YBAE-T42 vs SB-T42; YB-T42 vs SBAE-T42; YBAE-T42 vs YB-T42
**9-Octadecenal, (Z)-**	33.9	6.7541e-05	0.00061912	SBAE-T42 vs SB-T42; YBAE-T42 vs SB-T42; YB-T42 vs SBAE-T42; YBAE-T42 vs SBAE-T42
**Pentanoic acid**	28.016	0.00013543	0.00098149	SBAE-T42 vs SB-T42; YBAE-T42 vs SB-T42; YB-T42 vs SBAE-T42; YBAE-T42 vs YB-T42
**Butanoic acid, 3-methyl-**	27.611	0.00014276	0.00098149	YBAE-T42 vs SB-T42; YB-T42 vs SBAE-T42; YBAE-T42 vs SBAE-T42; YBAE-T42 vs YB-T42
**Tetradecanal**	26.66	0.00016201	0.00099005	SBAE-T42 vs SB-T42; YB-T42 vs SBAE-T42; YBAE-T42 vs SBAE-T42
**Butanoic acid**	25.222	0.00019767	0.0010872	SBAE-T42 vs SB-T42; YBAE-T42 vs SB-T42; YB-T42 vs SBAE-T42; YBAE-T42 vs YB-T42

**Phenylethyl Alcohol**	20.593	0.00040521	0.002026	SBAE-T42 vs SB-T42; YBAE-T42 vs SB-T42; YB-T42 vs SBAE-T42; YBAE-T42 vs YB-T42
**Octadecanal**	18.36	0.00060341	0.0027656	SBAE-T42 vs SB-T42; YB-T42 vs SBAE-T42; YBAE-T42 vs SBAE-T42
**2,4-Di-tert-butylphenol**	17.421	0.00072256	0.0029333	SBAE-T42 vs SB-T42; YBAE-T42 vs SB-T42; YB-T42 vs SBAE-T42; YBAE-T42 vs SBAE-T42
**2-Tetradecanone**	17.254	0.00074666	0.0029333	SBAE-T42 vs SB-T42; YB-T42 vs SBAE-T42; YBAE-T42 vs SBAE-T42
**Methyl Isobutyl Ketone**	15.937	0.00097744	0.0035839	YBAE-T42 vs SB-T42; YB-T42 vs SBAE-T42; YBAE-T42 vs YB-T42
**Gamma-Dodecalactone**	15.017	0.0011933	0.0040694	SBAE-T42 vs SB-T42; YB-T42 vs SBAE-T42; YBAE-T42 vs SBAE-T42
**Propanoic acid, 2-methyl-**	14.782	0.0012578	0.0040694	SBAE-T42 vs SB-T42; YBAE-T42 vs SB-T42; YB-T42 vs SBAE-T42; YBAE-T42 vs YB-T42
**p-Cresol**	14.276	0.001412	0.0043144	SBAE-T42 vs SB-T42; YBAE-T42 vs SB-T42; YB-T42 vs SBAE-T42; YBAE-T42 vs YB-T42
**8-Methylnonanoic acid**	13.917	0.0015358	0.0044457	SBAE-T42 vs SB-T42; YB-T42 vs SBAE-T42; YBAE-T42 vs SBAE-T42
**1-Propanol**	11.435	0.0028991	0.0078621	SBAE-T42 vs SB-T42; YBAE-T42 vs SB-T42; YB-T42 vs SBAE-T42; YBAE-T42 vs YB-T42
**Dodecanal**	11.309	0.0030019	0.0078621	SBAE-T42 vs SB-T42; YBAE-T42 vs SBAE-T42
**Phenol**	10.696	0.0035768	0.008942	YBAE-T42 vs SB-T42; YB-T42 vs SBAE-T42; YBAE-T42 vs YB-T42
**Benzaldehyde, 2,5-dimethyl-**	10.052	0.0043373	0.010372	YB-T42 vs SB-T42; YBAE-T42 vs YB-T42
**Hexanoic acid, ethyl ester**	9.7699	0.0047339	0.010848	YBAE-T42 vs SBAE-T42
**Cyclohexanecarboxylic acid**	9.4314	0.0052709	0.011596	YB-T42 vs SB-T42; YB-T42 vs SBAE-T42
**1H-Pyrrole-2,5-dione, 3-ethyl-**	9.2999	0.0054999	0.011634	SBAE-T42 vs SB-T42; YB-T42 vs SBAE-T42; YBAE-T42 vs SBAE-T42
**Undecane**	8.7574	0.0065871	0.013258	YB-T42 vs SB-T42; YBAE-T42 vs YB-T42
**Benzaldehyde**	8.6859	0.0067497	0.013258	SBAE-T42 vs SB-T42; YB-T42 vs SBAE-T42
**1H-Indole, 4-methyl-**	7.7279	0.0094999	0.018017	SBAE-T42 vs SB-T42; YB-T42 vs SB-T42; YBAE-T42 vs SB-T42
**Propanoic acid**	7.3238	0.011074	0.020303	SBAE-T42 vs SB-T42; YB-T42 vs SB-T42; YBAE-T42 vs SB-T42
**2-Tridecanone**	7.0796	0.012185	0.021618	YB-T42 vs SBAE-T42; YBAE-T42 vs SBAE-T42
**2-Pentadecanone**	6.3054	0.016759	0.028804	SBAE-T42 vs SB-T42; YBAE-T42 vs SBAE-T42
**2-Dodecanone**	5.6122	0.02281	0.038017	YB-T42 vs SBAE-T42
**3-Pentanone, 2-methyl-**	5.5327	0.023667	0.038285	YBAE-T42 vs YB-T42
