# Supplementary figures and images for: Gluten-Free Bread Enriched with Artichoke Leaf Extract In Vitro Exerted Antioxidant and Anti-Inflammatory Properties
Source: Antioxidants (Basel). 2023 Apr 1;12(4):845. doi: 10.3390/antiox12040845 (PMC10135093; doi:10.3390/antiox12040845)

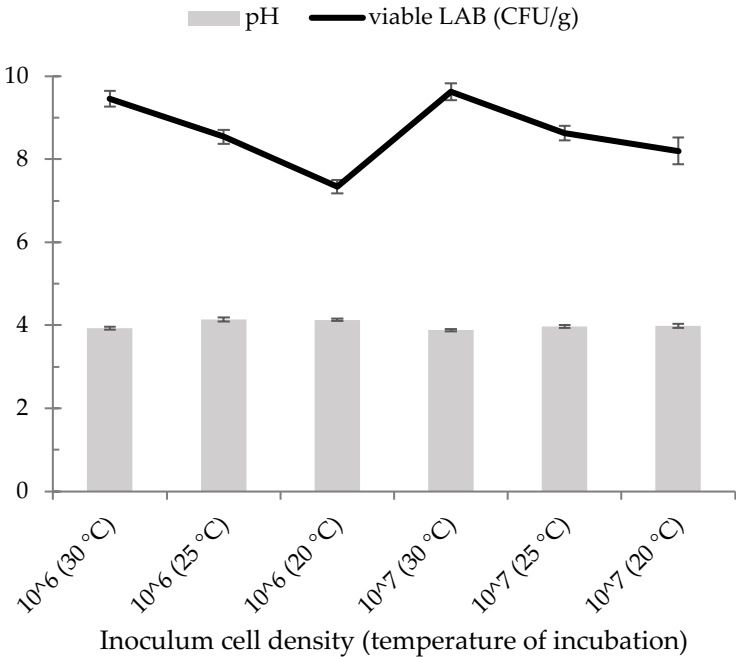

Supplement: Supplementary file 1 [file antioxidants-12-00845-s001.zip › Supp_FIG_S1_ArtBr_tIISD.pdf]

**A**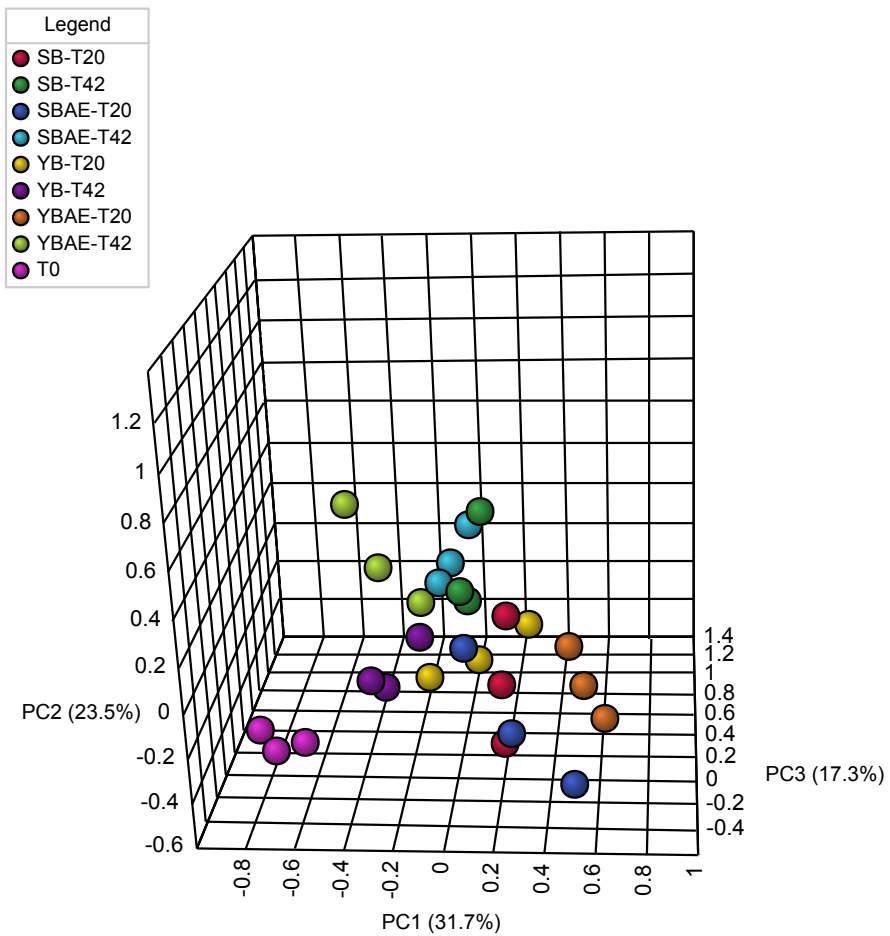**B**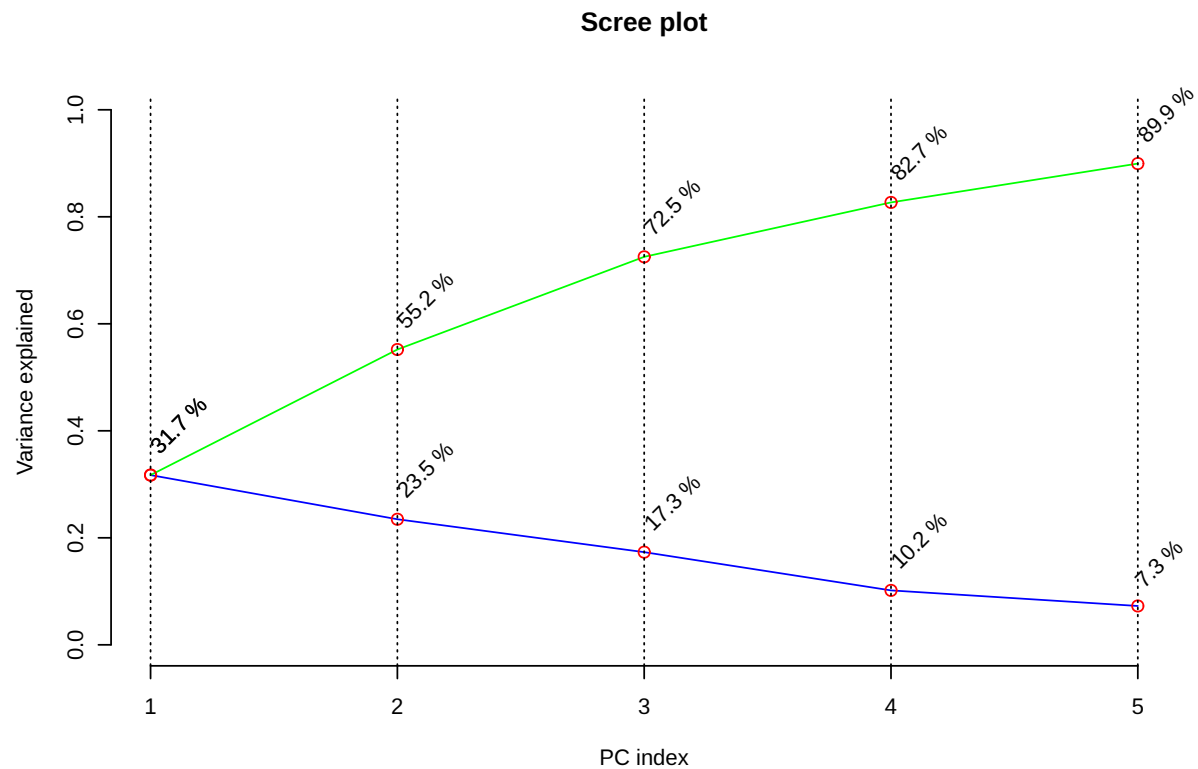

Supplement: Supplementary file 1 [file antioxidants-12-00845-s001.zip › Supp_FIG_S2_PCA.pdf]

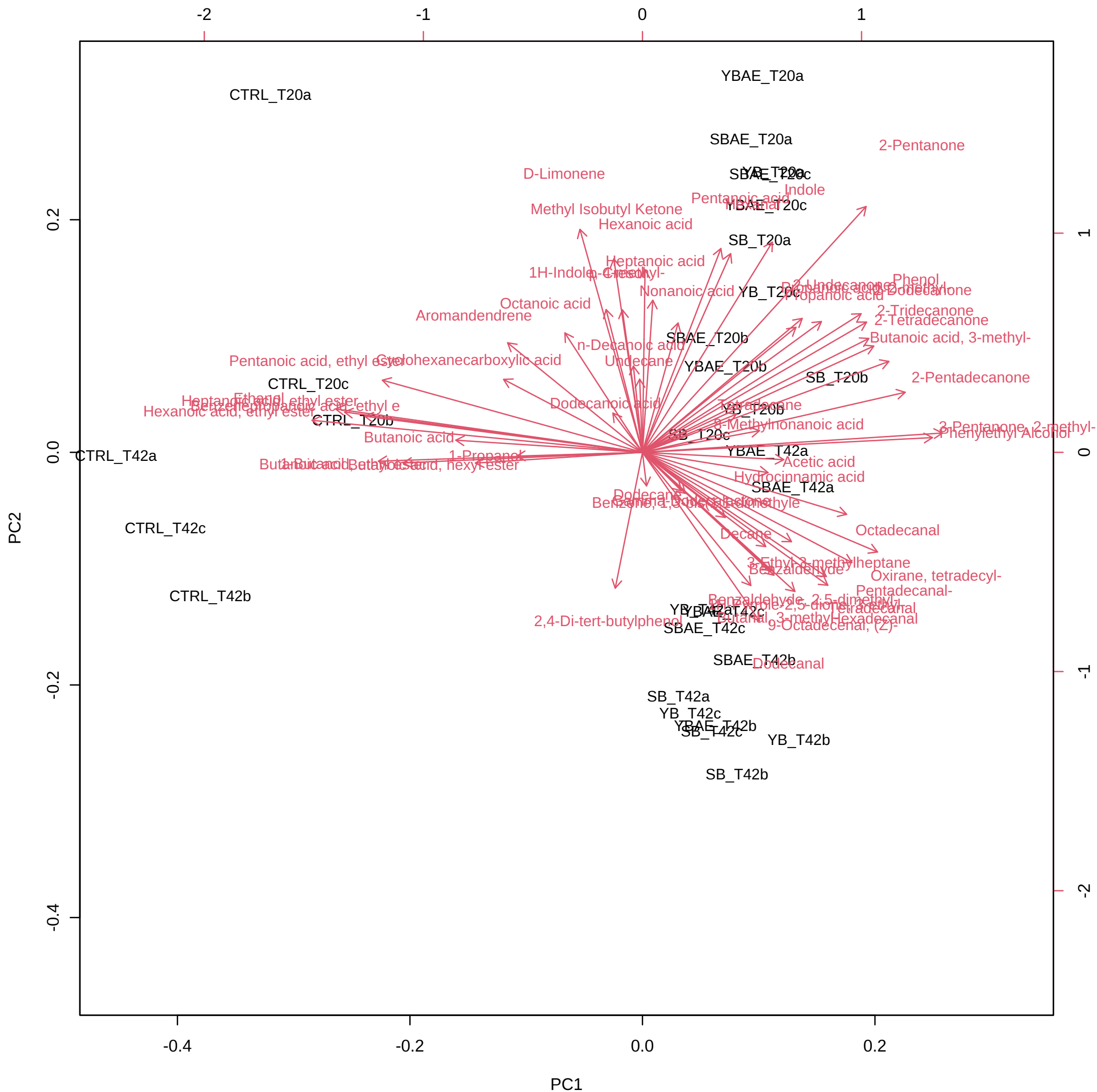

Supplement: Supplementary file 1 [file antioxidants-12-00845-s001.zip › Supp_FIG_S3_VOC_all.pdf]

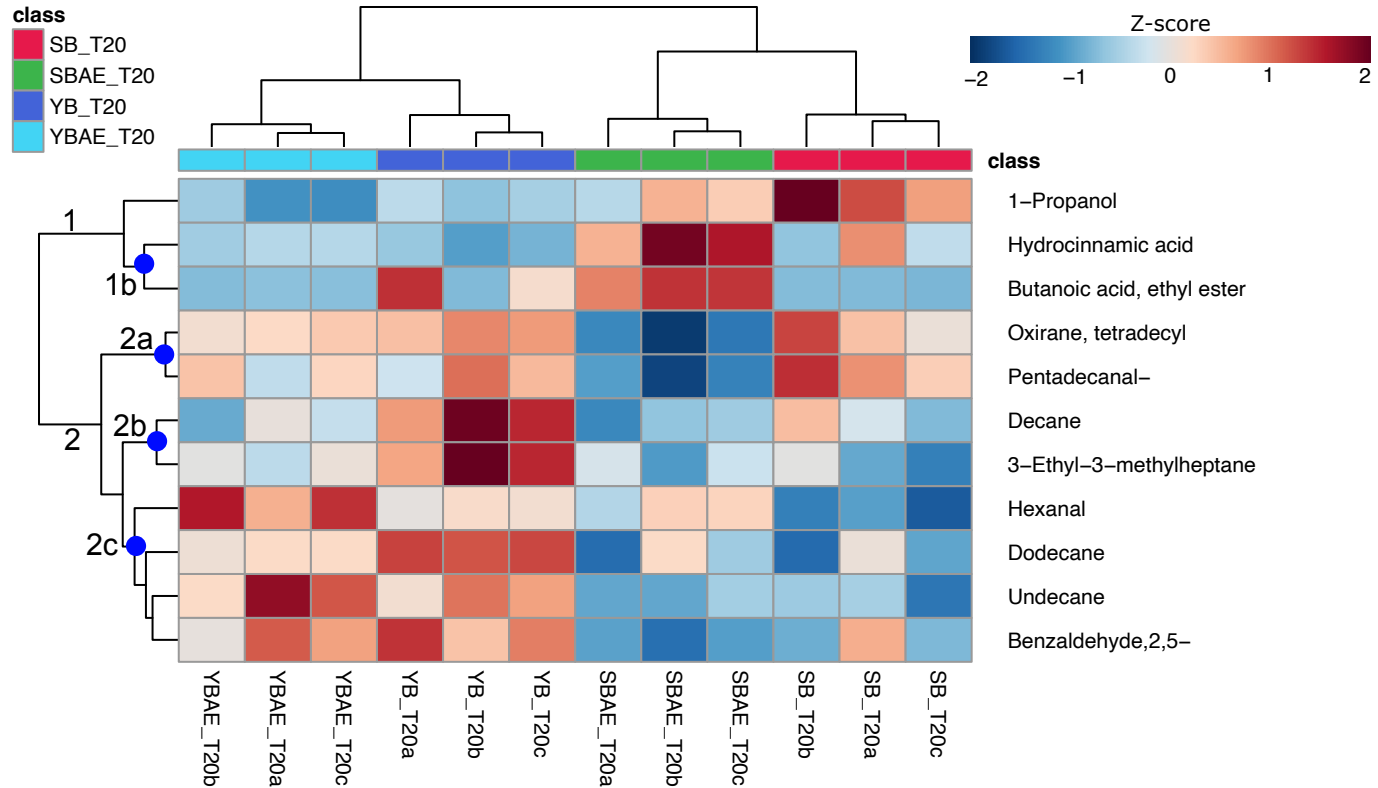

Supplement: Supplementary file 1 [file antioxidants-12-00845-s001.zip › Supp_FIG_S4_VOC_T20.pdf]

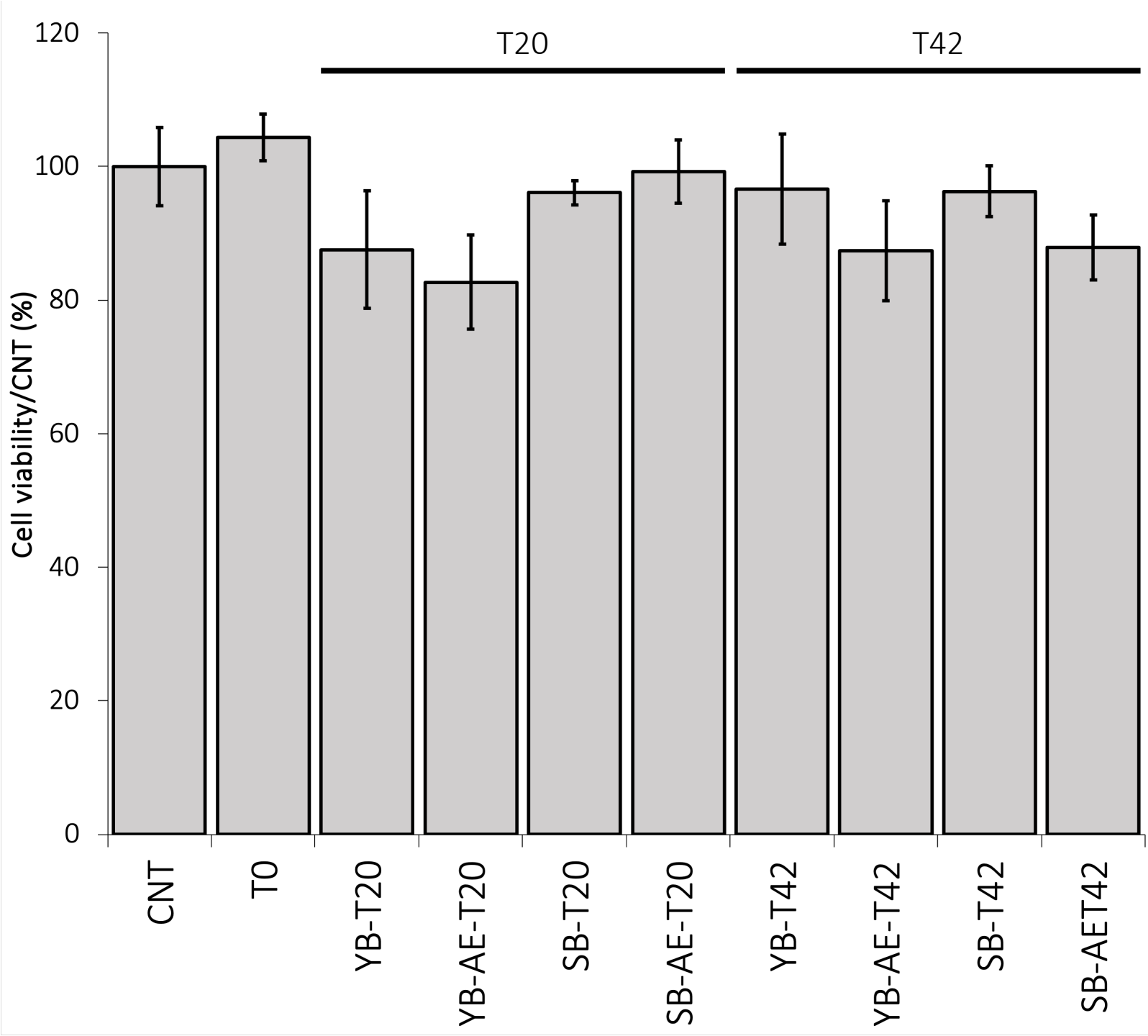

Supplement: Supplementary file 1 [file antioxidants-12-00845-s001.zip › Supp_FIG_S5_ArtBr_MTTassay.pdf]
